# Exploring the Social Media Discussion of Breast Cancer Treatment Choices: Quantitative Natural Language Processing Study

**DOI:** 10.2196/52886

**Published:** 2025-01-28

**Authors:** Daphna Y Spiegel, Isabel D Friesner, William Zhang, Travis Zack, Gianna Yan, Julia Willcox, Nicolas Prionas, Lisa Singer, Catherine Park, Julian C Hong

**Affiliations:** 1 Department of Radiation Oncology, Beth Israel Deaconess Medical Center, Harvard Medical School, 330 Brookline Ave, Boston, MA, 02215, United States, 1 6176672345; 2 Department of Radiation Oncology, University of California, San Francisco, San Francisco, CA, United States; 3 Bakar Computational Health Sciences Institute, University of California, San Francisco, San Francisco, CA, United States; 4 UCSF-UC Berkeley Joint Program in Computational Precision Health, San Francisco, CA, United States; 5 Department of Medicine, University of California, San Francisco, San Francisco, CA, United States

**Keywords:** breast cancer, social media, patient decision-making, natural language processing, breast conservation, mastectomy

## Abstract

**Background:**

Early-stage breast cancer has the complex challenge of carrying a favorable prognosis with multiple treatment options, including breast-conserving surgery (BCS) or mastectomy. Social media is increasingly used as a source of information and as a decision tool for patients, and awareness of these conversations is important for patient counseling.

**Objective:**

The goal of this study was to compare sentiments and associated emotions in social media discussions surrounding BCS and mastectomy using natural language processing (NLP).

**Methods:**

Reddit posts and comments from the Reddit subreddit r/breastcancer and associated metadata were collected using pushshift.io. Overall, 105,231 paragraphs across 59,416 posts and comments from 2011 to 2021 were collected and analyzed. Paragraphs were processed through the Apache Clinical Text Analysis Knowledge Extraction System and identified as discussing BCS or mastectomy based on physician-defined Systematized Nomenclature of Medicine Clinical Terms (SNOMED CT) concepts. Paragraphs were analyzed with a VADER (Valence Aware Dictionary for Sentiment Reasoning) compound sentiment score (ranging from −1 to 1, corresponding to negativity or positivity) and GoEmotions scores (0‐1) corresponding to the intensity of 27 different emotions and neutrality.

**Results:**

Of the 105,231 paragraphs, there were 7306 (6.94% of those analyzed) paragraphs mentioning BCS and mastectomy (2729 and 5476, respectively). Discussion of both increased over time, with BCS outpacing mastectomy. The median sentiment score for all discussions analyzed in aggregate became more positive over time. In specific analyses by topic, positive sentiments for discussions with mastectomy mentions increased over time; however, discussions with BCS-specific mentions did not show a similar trend and remained overall neutral. Compared to BCS, conversations about mastectomy tended to have more positive sentiments. The most commonly identified emotions included neutrality, gratitude, caring, approval, and optimism. Anger, annoyance, disappointment, disgust, and joy increased for BCS over time.

**Conclusions:**

Patients are increasingly participating in breast cancer therapy discussions with a web-based community. While discussions surrounding mastectomy became increasingly positive, BCS discussions did not show the same trend. This mirrors national clinical trends in the United States, with the increasing use of mastectomy over BCS in early-stage breast cancer. Recognizing sentiments and emotions surrounding the decision-making process can facilitate patient-centric and emotionally sensitive treatment recommendations.

## Introduction

Early-stage breast cancer has the complex challenge of carrying a favorable prognosis with multiple treatment options, including breast-conserving surgery (BCS) or mastectomy. Treatment decisions are therefore driven by patient preferences, making information gathering and decision analysis critical. Multiple randomized trials have shown that locoregional recurrence and survival rates are similar with breast-conserving therapy (BCT) or mastectomy, with recent data even suggesting improved survival with BCT [1,2]. Nonetheless, trends indicate that women with early-stage, nonhereditary breast cancer are increasingly choosing mastectomy [3].

Many factors contribute to patient decision-making for cancer therapy, including the growing influence of social media. Several previous studies have investigated the use of online forums and social media by patients with breast cancer [4-8]. As many as 77% of patients with breast cancer cite the internet as their primary information source [9]. Additionally, patients who are frequent users of online communication and social media tools experience increased decision-satisfaction [10]. Additionally, large language models, including ChatGPT, are being increasingly used by patients for medical decision making. As social media data, including that from Reddit, are used to train these models, these discussions are relevant to the information that patients receive [11,12].

There are limited data characterizing social media conversations surrounding the decision regarding BCS or mastectomy, which is important to understand to gain insights into national trends in the United States and inform the counseling process. We applied sentiment and emotion analyses with natural language processing (NLP) approaches to a popular breast cancer online community to compare the sentiments and associated emotions around conversations of BCS and mastectomy.

## Methods

### Data Source

The moderated Reddit subreddit r/breastcancer was created on December 3, 2011, and is self-described as “a support and information group for people who have been diagnosed with breast cancer and for their caregivers and loved ones.” As of January 2023, it had 13,700 subscribed members. Out of all internet users, 8% of men and 4% of women used Reddit; of those, 11% were aged 18‐29 years, 7% were aged 30‐39 years, 2% were aged 50‐64 years, and 2% were aged 65+ years [13]. We selected Reddit for social and technical reasons, as it is anonymous, public, open, and interaction-centric. Reddit text is also frequently used to train NLP algorithms, reducing concerns about model applicability.

All posts, comments, and metadata from r/breastcancer from 2011 to 2021 were collected using pushshift.io [14]. Pushshift.io is a public social media archiving platform with real-time Reddit data for social media research. As of March 20, 2024, it has 908 citations according to Google Scholar. Data from Pushshift.io were accessed on February 4, 2022.

### Data Preprocessing and Topic Identification

These posts and comments were separated into paragraphs based on line breaks to separate topics for analysis. We applied the Apache cTAKES (Clinical Text Analysis Knowledge Extraction System) v. 4.0.0 default clinical pipeline to identify mentions of BCS- or mastectomy-related terms mapped to the Systematized Nomenclature of Medicine Clinical Terms (SNOMED CT). cTAKES is an NLP software with multiple parts designed to process clinical free text. It had a sentence boundary detector, tokenizer, normalizer, part-of-speech tagger, shallow parser, and a named entity recognition annotator with negation. SNOMED keywords related to BCS or mastectomy were identified by a physician author (DYS). Examples of SNOMED words or phrases used in this analysis include *simple mastectomy*, *bilateral mastectomy*, and *modified radical mastectomy* as mastectomy keywords and *lumpectomy*, *excision biopsy*, and *segmental mastectomy* as BCS keywords. Analyses of paragraphs were based on references to BCS or mastectomy, nonexclusively. Paragraphs containing mentions of both were attributed to both treatments in the analysis.

### Sentiment Analysis

Paragraphs (as a whole) were analyzed using VADER (Valence Aware Dictionary for Sentiment Reasoning) to generate compound sentiment scores from −1 to 1 (negative to positive). VADER is a popular sentiment analysis model trained on social media text, with performance comparable to more complex approaches and advantages of computational efficiency, explainability, and domain agnosticism or generalizability [12].

### Emotion Classification

GoEmotions is the largest human-annotated dataset of fine-grained emotions, with 58,000 Reddit comments labeled for 27 emotions and neutrality [11]. We applied a publicly available BERT (Bidirectional Encoder Representations from Transformers) model from Google Research [15]. GoEmotions generates a score from 0 to 1 based on the intensity of the detected emotion. A paragraph was considered to express an emotion if its score was >0.5, as used in intensity annotations in the original GoEmotions benchmark studies [11].

Statistics were aggregated longitudinally for summary statistics per year. Years 2011 to 2014 had less than 100 posts discussing BCS or mastectomy; years 2011‐2017 were pooled due to limited sample size. Sentiment scores were compared between BCS and mastectomy using a 2-tailed Student *t* test. Emotions across treatments were visualized using radar charts.

### Ethical Considerations

The study data used in this analysis are anonymous, public, and open source. Therefore, there is minimal risk to performing these analyses. This study was approved by the University of San Francisco institutional review board, where the data collection and analyses were performed (IRB #21‐35353).

## Results

A total of 59,416 posts with 105,231 paragraphs on r/breastcancer, which were posted by 5845 users, were analyzed. There were 2729 mentions of BCS and 5476 mentions of mastectomy, nonexclusively. Post volume increased over time (5282 in 2011‐2017 to 44,235 in 2021). Words per paragraph had a slight increase over time, from a median of 28 (IQR 11‐53) words per line in 2011‐2017 to 26 (IQR 11‐49) in 2018, 28 (IQR 12‐50) in 2019, 30 (IQR 14‐55) in 2020, and 30 (IQR 14‐54) in 2021. The median number of comments per user was 2 (IQR 1‐7).

Discussion of both BCS and mastectomy increased over time, but BCS outpaced mastectomy, with an increasing ratio of BCS to mastectomy mentions (0.312 in 2011‐2017 to 0.583 in 2021). The median (IQR) sentiment score for all discussions became more positive annually: 0 (IQR –0.361 to 0.624) in 2011‐2017 to 0.288 (–0.223 to 0.701) in 2021. Positive sentiments for mastectomy generally increased over time: median of 0 (IQR –0.599 to 0.726) in 2011‐2017 to a median of 0.178 (IQR –0.511 to 0.73) in 2021. Similarly, the proportion of positive mastectomy-related discussions increased annually from 48.1% (151/314) in 2011‐2017 to 53.3% (1107/2076) in 2021. Discussion of BCS remained neutral—median 0 (IQR –0.494 to 0.523) to median 0.039 (IQR –0.511 to 0.642)—and the proportion of positive BCS discussions did not show a similar trend year-to-year: 43.9% (43/98) in 2011-2017 to 52.3% (139/266) in 2019, and stable in 2021 to 50.7% (614/1211). Compared to BCS, conversations about mastectomy were more positive (*P*=.02), driven primarily by differences in 2021—median 0.178 (IQR –0.511 to 0.73) for mastectomy vs median 0.039 (IQR –0.511 to 0.642) for BCS, with *P*=.049.

**Figure 1.**
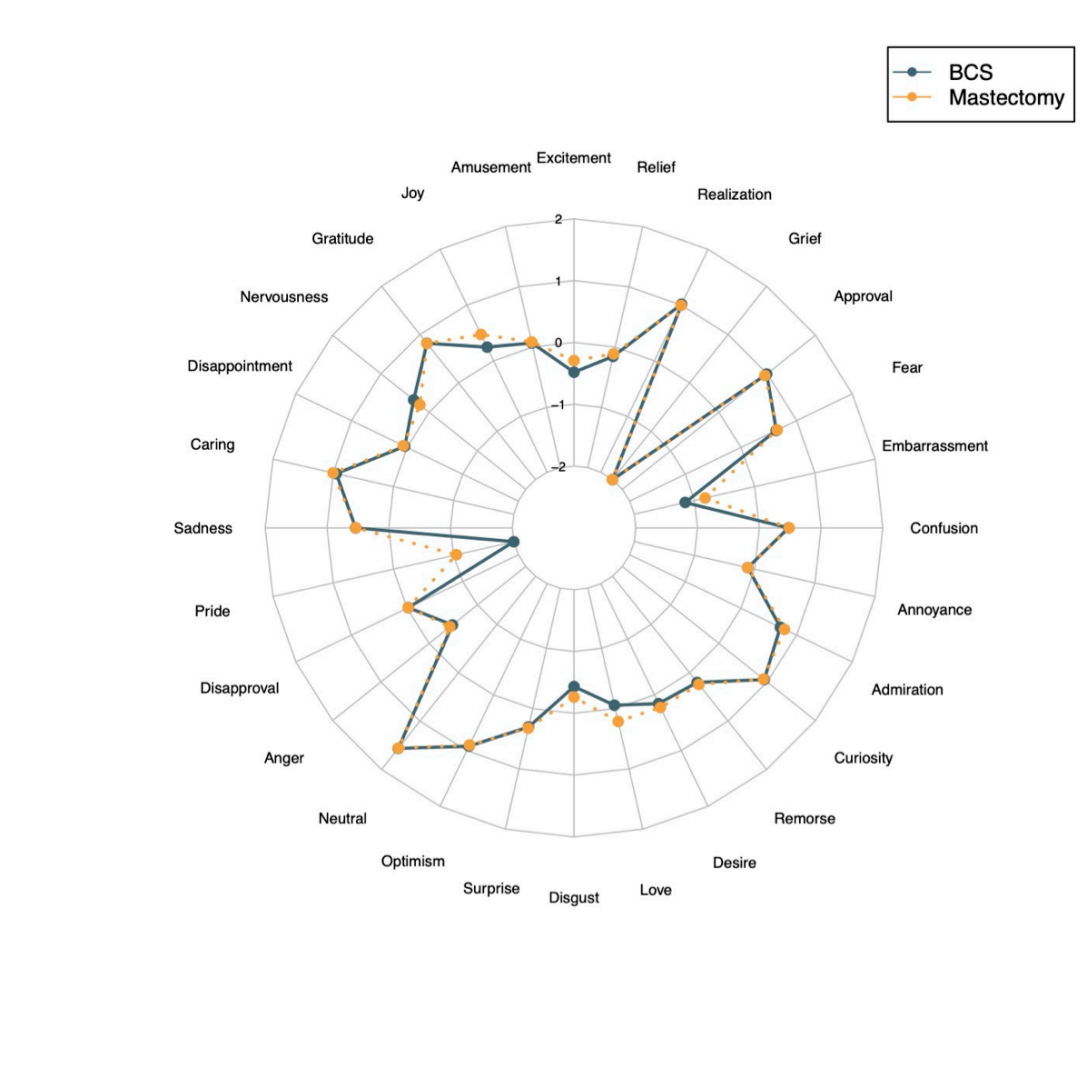
Radar chart of average emotion score across breast-conserving surgery (BCS) and mastectomy posts, respectively.

The most common emotions across r/breastcancer were neutrality, gratitude, caring, approval, and optimism. The most common emotions for both BCS and mastectomy were similar: neutrality (BCS: 1001/2729, 36.68%; mastectomy: 1973/5476, 36.03%), caring (BCS: 242/2729, 8.87%; mastectomy: 547/5476, 9.99%), approval (BCS: 267/2729, 9.78%; mastectomy: 492/5476, 8.98%), realization (BCS: 284/2729, 10.41%; mastectomy: 542/5476, 9.9%), and curiosity (BCS: 237/2729, 8.68%; mastectomy: 459/5476, 8.38%) (Figure 1). Six emotions increased over time for all posts: approval (2011‐2017: 354/5282, 6.7%; 2021: 4176/44,235, 9.44%), amusement (2011‐2017: 34/5282, 0.64%; 2021: 677/44,235, 1.53%), desire (2011‐2017: 37/5282, 0.7%; 2021: 552/44,235, 1.25%), disappointment (2011‐2017: 64/5282, 1.21%; 2021: 787/44,235, 1.78%), excitement (2011‐2017: 23/2729, 0.44%; 2021: 477/44,235, 1.08%), and realization (2011‐2017: 259/5282, 4.9%; 2021: 2902/44,235, 6.56%). Fear (2011‐2017: 203/5282, 3.84%; 2021: 1223/44,235, 2.76%) and neutrality (2011‐2017: 2144/5282, 40.59%; 2021: 14,514/44,235, 32.81%) decreased over time (Table 1).

**Table 1.** Comparison of emotion trends.^a^

Emotion		All messages, n/N (%)	Breast conservation, n/N (%)	Mastectomy, n/N (%)
**Amusement**				
	Overall	1379/105,231 (1.31)	32/2729 (1.17)	67/5476 (1.22)
	2011‐2017	34/5282 (0.64)	0/98 (0)	2/314 (0.64)
	2018	59/8144 (0.72)	3/174 (1.72)	3/410 (0.73)
	2019	109/11,943 (0.91)	1/266 (0.38)	10/673 (1.49)
	2020	500/35,627 (1.4)	16/980 (1.63)	32/2003 (1.6)
	2021	677/44,235 (1.53)	12/1211 (0.99)	20/2076 (0.96)
**Anger**				
	Overall	762/105,231 (0.72)	9/2729 (0.33)	20/5476 (0.37)
	2011‐2017	48/5282 (0.91)	0/98 (0)	1/314 (0.32)
	2018	65/8144 (0.8)	0/174 (0)	2/410 (0.49)
	2019	68/11,943 (0.57)	0/266 (0)	1/673 (0.15)
	2020	245/35,627 (0.69)	4/980 (0.41)	5/2003 (0.25)
	2021	336/44,235 (0.76)	5/1211 (0.41)	11/2076 (0.53)
**Annoyance**				
	Overall	1536/105,231 (1.46)	21/2729 (0.77)	42/5476 (0.77)
	2011‐2017	66/5282 (1.25)	0/98 (0)	3/314 (0.96)
	2018	105/8144 (1.29)	0/174 (0)	1/410 (0.24)
	2019	177/11,943 (1.48)	1/266 (0.38)	7/673 (1.04)
	2020	487/35,627 (1.37)	5/980 (0.51)	12/2003 (0.6)
	2021	701/44,235 (1.58)	15/1211 (1.24)	19/2076 (0.92)
**Approval**				
	Overall	9515/105,231 (9.04)	267/2729 (9.78)	492/5476 (8.98)
	2011‐2017	354/5282 (6.7)	9/98 (9.18)	19/314 (6.05)
	2018	647/8144 (7.94)	15/174 (8.62)	41/410 (10)
	2019	1010/11,943 (8.46)	23/266 (8.65)	60/673 (8.92)
	2020	3328/35,627 (9.34)	979/980 (9.9)	184/2003 (9.19)
	2021	4176/44,235 (9.44)	123/1211 (10.16)	188/2076 (9.06)
**Caring**				
	Overall	11,640/105,231 (11.06)	242/2729 (8.87)	547/5476 (9.99)
	2011‐2017	562/5282 (10.64)	9/98 (9.18)	39/314 (12.42)
	2018	1062/8144 (13.04)	23/174 (13.22)	61/410 (14.88)
	2019	1353/11,943 (11.33)	21/266 (7.89)	50/673 (7.43)
	2020	3958/35,627 (11.11)	97/980 (9.9)	199/2003 (9.94)
	2021	4705/44,235 (10.64)	92/1211 (7.6)	198/2076 (9.54)
**Curiosity**				
	Overall	6973/105,231 (6.63)	237/2729 (8.68)	459/5476 (8.38)
	2011‐2017	429/5282 (8.12)	11/98 (11.22)	35/314 (11.15)
	2018	567/8144 (6.93)	14/174 (8.05)	28/410 (6.83)
	2019	724/11,943 (6.06)	24/266 (9.02)	44/673 (6.54)
	2020	2263/35,627 (6.35)	84/980 (8.57)	167/2003 (8.34)
	2021	2993/44,235 (6.77)	104/1211 (8.59)	185/2076 (8.91)
**Desire**				
	Overall	1139/105,231 (1.08)	39/2729 (1.43)	92/5476 (1.68)
	2011‐2017	37/5282 (0.7)	1/98 (1.02)	3/314 (0.96)
	2018	72/8144 (0.88)	3/174 (1.72)	6/410 (1.46)
	2019	107/11,943 (0.9)	3/266 (1.13)	8/673 (1.19)
	2020	371/35,627 (1.04)	17/980 (1.73)	38/2003 (1.9)
	2021	552/44,235 (1.25)	15/1211 (1.24)	37/2076 (1.78)
**Disappointment**				
	Overall	1696/105,231 (1.61)	30/2729 (1.1)	64/5476 (1.17)
	2011‐2017	64/5282 (1.21)	0/98 (0)	3/314 (0.96)
	2018	106/8144 (1.3)	1/174 (0.57)	3/410 (0.73)
	2019	174/11,943 (1.46)	2/266 (0.75)	7/673 (1.04)
	2020	565/35,627 (1.59)	9/980 (0.92)	24/2003 (1.2)
	2021	787/44,235 (1.78)	18/1211 (1.49)	27/2076 (1.3)
**Disgust**				
	Overall	630/105,231 (0.6)	10/2729 (0.37)	30/5476 (0.55)
	2011‐2017	20/5282 (0.38)	0/98 (0)	0/314 (0)
	2018	35/8144 (0.43)	0/174 (0)	5/410 (1.22)
	2019	81/11,943 (0.68)	1/266 (0.38)	7/673 (1.04)
	2020	193/35,627 (0.54)	4/980 (0.41)	9/2003 (0.45)
	2021	301/44,235 (0.68)	5/1211 (0.41)	9/2076 (0.43)
**Embarrassment**				
	Overall	171/105,231 (0.16)	2/2729 (0.07)	8/5476 (0.15)
	2011‐2017	9/5282 (0.17)	0/98 (0)	0/314 (0)
	2018	3/8144 (0.04)	0/174 (0)	1/410 (0.24)
	2019	19/11,943 (0.16)	0/266 (0)	0/673 (0)
	2020	55/35,627 (0.15)	0/980 (0)	1/2003 (0.05)
	2021	85/44,235 (0.19)	2/1211 (0.17)	6/2076 (0.29)
**Excitement**				
	Overall	933/105,231 (0.89)	9/2729 (0.33)	28/5476 (0.51)
	2011‐2017	23/5282 (0.44)	0/98 (0)	2/314 (0.64)
	2018	37/8144 (0.45)	0/174 (0)	2/410 (0.49)
	2019	91/11,943 (0.76)	0/266 (0)	4/673 (0.59)
	2020	305/35,627 (0.86)	5/980 (0.51)	10/2003 (0.5)
	2021	477/44,235 (1.08)	4/1211 (0.33)	10/2076 (0.48)
**Fear**				
	Overall	3223/105,231 (3.06)	118/2729 (4.32)	247/5476 (4.51)
	2011‐2017	203/5282 (3.84)	2/98 (2.04)	18/314 (5.73)
	2018	283/8144 (3.47)	4/174 (2.3)	26/410 (6.34)
	2019	396/11,943 (3.32)	10/266 (3.76)	26/673 (3.86)
	2020	1118/35,627 (3.14)	50/980 (5.1)	95/2003 (4.74)
	2021	1223/44,235 (2.76)	52/1211 (4.29)	82/2076 (3.95)
**Joy**				
	Overall	2392/105,231 (2.27)	48/2729 (1.76)	162/5476 (2.96)
	2011‐2017	97/5282 (1.84)	0/98 (0)	8/314 (2.55)
	2018	136/8144 (1.67)	2/174 (1.15)	14/410 (3.41)
	2019	234/11,943 (1.96)	4/266 (1.5)	20/673 (2.97)
	2020	786/35,627 (2.21)	18/980 (1.84)	60/2003 (3)
	2021	1139/44,235 (2.57)	24/1211 (1.98)	60/2076 (2.89)
**Love**				
	Overall	2095/105,231 (1.99)	24/2729 (0.88)	89/5476 (1.63)
	2011‐2017	96/5282 (1.82)	0/98 (0)	9/314 (2.87)
	2018	113/8144 (1.39)	0/174 (0)	9/410 (2.2)
	2019	178/11,943 (1.49)	3/266 (1.13)	7/673 (1.04)
	2020	678/35,627 (1.9)	7/980 (0.71)	31/2003 (1.55)
	2021	1030/44,235 (2.33)	14/1211 (1.16)	33/2076 (1.59)
**Nervousness**				
	Overall	1218/105,231 (1.16)	57/2729 (2.09)	85/5476 (1.55)
	2011‐2017	68/5282 (1.29)	1/98 (1.02)	5/314 (1.59)
	2018	109/8144 (1.34)	2/174 (1.15)	2/410 (0.49)
	2019	155/11,943 (1.3)	5/266 (1.88)	6/673 (0.89)
	2020	404/35,627 (1.13)	22/980 (2.24)	29/2003 (1.45)
	2021	482/44,235 (1.09)	27/1211 (2.23)	43/2076 (2.07)
**Neutral**				
	Overall	36,373/105,231 (34.56)	1001/2729 (36.68)	1973/5476 (36.03)
	2011‐2017	2144/5282 (40.59)	39/98 (39.8)	116/314 (36.94)
	2018	3166/8144 (38.8)	66/174 (37.93)	148/410 (36.1)
	2019	4502/11,943 (37.7)	102/266 (38.35)	288/673 (42.79)
	2020	12,047/35,627 (33.81)	336/980 (34.29)	689/2003 (34.4)
	2021	14,514/44,235 (32.81)	458/1211 (37.82)	732/2076 (35.26)
**Pride**				
	Overall	91/105,231 (0.09)	0/2729 (0)	5/5476 (0.09)
	2011‐2017	5/5282 (0.09)	0/98 (0)	0/314 (0)
	2018	4/8144 (0.05)	0/174 (0)	1/410 (0.24)
	2019	9/11,943 (0.08)	0/266 (0)	0/673 (0)
	2020	34/35,627 (0.1)	0/980 (0)	2/2003 (0.1)
	2021	39/44,235 (0.09)	0/1211 (0)	2/2076 (0.1)
**Realization**				
	Overall	6602/105,231 (6.27)	284/2729 (10.41)	542/5476 (9.9)
	2011‐2017	259/5282 (4.9)	10/98 (10.2)	27/314 (8.6)
	2018	438/8144 (5.38)	19/174 (10.92)	39/410 (9.51)
	2019	744/11,943 (6.23)	29/266 (10.9)	70/673 (10.4)
	2020	2259/35,627 (6.34)	100/980 (10.2)	198/2003 (9.89)
	2021	2902/44,235 (6.56)	126/1211 (10.4)	208/2076 (10.02)

a Comparison of emotion trends overall and over time across all paragraphs and separated by breast conservation or mastectomy, nonexclusively, in r/breastcancer.

Five emotions became increasingly prevalent for BCS, although they were rare: anger (2011‐2017: 0/98, 0%; 2021: 5/1211, 0.41%), annoyance (2011‐2017: 0/98, 0%; 2021: 15/1211, 1.24%), disappointment (2011‐2017: 0/98, 0%; 2021: 18/1211, 1.49%), disgust (2011‐2017: 0/98, 0%; 2021: 5/1211, 0.41%), and joy (2011‐2017: 0/98, 0%; 2021: 24/1211, 1.98%). No emotions showed a consistent trend for mastectomy-related posts (Table 1). Additionally, after 2017, realization and nervousness were more common for BCS than mastectomy annually. Realization, approval, and caring were the most strongly expressed emotions across both BCS (top decile scores: 0.52, 0.48, and 0.43, respectively) and mastectomy (top decile scores: 0.49, 0.41, and 0.5), with breast conservation being more associated with optimism (top decile score: 0.33).

## Discussion

### Principal Findings

Building upon past research in sentiment analysis of online discussions about breast cancer [4,5,16], NLP identified differences in social media discussions across BCS and mastectomy, reflecting trends reported clinically. Compared to previous studies [4,5,16] conducting sentiment analyses of online forums discussing cancer, our work focused specifically on surgical management options for patients with breast cancer. Our distinct NLP approaches identified that discussions surrounding mastectomy became increasingly positive over time, corresponding with concordant emotions. These findings are consistent with multiple studies that have found a growing trend of patients with early-stage breast cancer choosing mastectomy over BCS [3,17]. While it is not feasible to determine the reason for the observed increase in positive sentiments for mastectomy mentions based on the data available in this study, it does indicate a parallel with real-world patient decision-making.

Discussion in this breast cancer–specific forum increased substantially over time, confirming that patients are increasingly using social media as a resource. BCS and mastectomy-related posting increased, emphasizing trends in content-specific information. In a recent survey study, patients reported that their cancer diagnosis prompted them to join social media platforms, and over 80% of respondents reported using social media to gather information online [9]. The predominance of objectivity (neutrality emotion) and informative (realization and curiosity) emotions supports these findings.

Evidence surrounding treatment choice for early-stage breast cancer suggests the decision to pursue mastectomy over BCS is often driven by fear of recurrence and secondary cancers [18]. Our application of NLP identifies this in the online setting, with BCS posts more likely to express nervousness. Negative emotions such as anger, annoyance, disappointment, and disgust also became increasingly prevalent over time in BCS posts.

This study is limited by confounders. While VADER and GoEmotions are specifically developed for social media text and based on complementary approaches, they also may reflect inaccuracies and biases based on limitations in their training. Moreover, sentiments cannot be explicitly attributed to the topics themselves, but rather to the paragraphs associated with specific treatments. Nevertheless, these paragraphs likely reflect related discussions around each of these treatments or some aspect of related care.

These findings provide unique insight into patient decision-making. Social media reflects real-time discussions in a natural setting with less filtered discussion of patient concerns and experiences. Recognizing the sentiments and emotions expressed surrounding the treatment, the decision-making process can help clinicians create patient-centric recommendations.

### Conclusion

As social media becomes more pervasive, patients are increasingly discussing options for breast cancer therapy online. NLP can characterize these candid online patient discussions at scale and help clinicians identify barriers to treatment decisions and strengthen counseling for patients. Additional studies will be required to see if ongoing social media sentiment trends continue to track patient decisions.

## Abbreviations

BCS: breast-conserving surgery
BCT: breast-conserving therapy
BERT: Bidirectional Encoder Representations from Transformers
cTAKES: Clinical Text Analysis Knowledge Extraction System
NLP: natural language processing
SNOMED CT: Systematized Nomenclature of Medicine Clinical Terms
VADER: Valence Aware Dictionary for Sentiment Reasoning
